# Hydrogen Peroxide Electrosynthesis in a Strong Acidic Environment Using Cationic Surfactants

**DOI:** 10.1021/prechem.3c00096

**Published:** 2024-02-03

**Authors:** Zachary Adler, Xiao Zhang, Guangxia Feng, Yaping Shi, Peng Zhu, Yang Xia, Xiaonan Shan, Haotian Wang

**Affiliations:** † Rice University, Department of Chemical and Biomolecular Engineering, Houston, Texas 77005, United States; ‡ University of Houston, Department of Electrical and Computer Engineering, Houston, Texas 77004, United States; § Rice University, Department of Chemistry, Houston, Texas 77005, United States; ∥ Rice University, Department of Materials Science and Nanoengineering, Houston, Texas 77005, United States

**Keywords:** electrocatalysis, hydrogen peroxide, electrosynthesis, oxygen reduction reaction, interfacial engineering, surfactant, CTAB

## Abstract

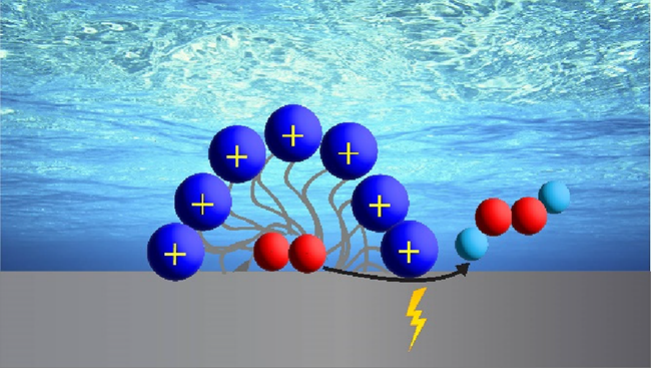

The two-electron oxygen reduction reaction (2e^–^-ORR) can be exploited for green production of hydrogen peroxide (H_2_O_2_), but it still suffers from low selectivity in an acidic electrolyte when using non-noble metal catalysts. Here, inspired by biology, we demonstrate a strategy that exploits the micellization of surfactant molecules to promote the H_2_O_2_ selectivity of a low-cost carbon black catalyst in strong acid electrolytes. The surfactants near the electrode surface increase the oxygen solubility and transportation, and they provide a shielding effect that displaces protons from the electric double layer (EDL). Compared with the case of a pure acidic electrolyte, we find that, when a small number of surfactant molecules were added to the acid, the H_2_O_2_ Faradaic efficiency (FE) was improved from 12% to 95% H_2_O_2_ under 200 mA cm^–2^, suggesting an 8-fold improvement. Our in situ surface enhanced Raman spectroscopy (SERS) and optical microscopy (OM) studies suggest that, while the added surfactant reduces the electrode’s hydrophobicity, its micelle formation could promote the O_2_ gas transport and its hydrophobic tail could displace local protons under applied negative potentials during catalysis, which are responsible for the improved H_2_O_2_ selectivity in strong acids.

## Introduction

Hydrogen peroxide (H_2_O_2_), as one of the most important chemicals, is widely applied in paper and pulp manufacturing, disinfection, water treatment, and chemical synthesis.^1−3^ The production and use of H_2_O_2_ has been increasing over the past several years,^4^ and it has gained new attention due to the COVID-19 pandemic. Currently, H_2_O_2_ is mostly manufactured via the anthraquinone cycling process.^2,3^ The cons of the anthraquinone process include high volumes of organic solvent and expensive noble metal catalyst utilization, large carbon footprint, expensive postsynthesis separations, and the installation of heavy infrastructure in centralized settings. In the past few decades, research has unveiled a more attractive method of H_2_O_2_ production, the electrosynthesis of H_2_O_2_ via the 2e^–^-ORR.^1,5−9^ In the electrosynthesis process, renewable electricity can be employed to drive the reactions at ambient temperature, the emissions are environmentally benign, and the H_2_O_2_ can be produced in a decentralized manner without further separation processes. This 2e^–^ process differs from the traditional 4e^–^-ORR process, which generates H_2_O in H_2_/O_2_ fuel cells. Catalyst selection is therefore important in order to steer the reaction toward the 2e^–^ path.^6^ Many early works focused on noble metal and noble metal alloy catalysts,^10−13^ though due to their high costs, more recent works have explored carbon-based materials as efficient catalysts for the 2e^–^ ORR, e.g., graphene, carbon nanotube (CNT), and amorphous carbon.^14−16^

Aside from catalyst selection, electrolyte selection is another critical factor in conducting H_2_O_2_ electrosynthesis. Alkaline electrolytes are preferred due to the superior selectivity toward H_2_O_2_ especially when using carbon-based catalysts,^7,11,12,14,15^ but high-pH solutions also promote the H_2_O_2_ decomposition into HO_2_^–^.^5,17,18^ In acidic solutions, the high concentration of protons at the electrode/electrolyte interface can easily lead to the hydrogen evolution reaction (HER) as well as the over-reduction of H_2_O_2_ to H_2_O, both of which are undesirable since they decrease the H_2_O_2_ selectivity. These phenomena lead to a dilemma in which acid can help stabilize the generated H_2_O_2_, but alkaline environments can be more selective to H_2_O_2_ synthesis. In addition, the type of ion-exchange membrane is another factor that contributes to the application of acidic electrolyte. Proton-exchange membranes (PEMs) are required in acidic environments, and the most common PEM, Nafion, is very stable and commercially available. In contrast, anion-exchange membranes (AEMs), employed in alkaline environments, are not as reliable as PEMs. Some papers have reported high selectivity >90% FE H_2_O_2_ in acid electrolytes but only under current densities of a few milliamperes per centimeter squared.^10,13,19,20^ For industrial applications, however, high current densities of several hundreds of milliamperes per centimeter square are necessary. Generally, higher current densities require larger overpotentials, at which the increased negative charge applied at the cathode will continuously attract protons to the surface, and thus make the system more susceptible to HER and the 4e^–^-ORR. The selectivity–stability dilemma compels us to seek ways to influence the triple phase, gas–electrode–electrolyte, interface.

**Figure 1:**
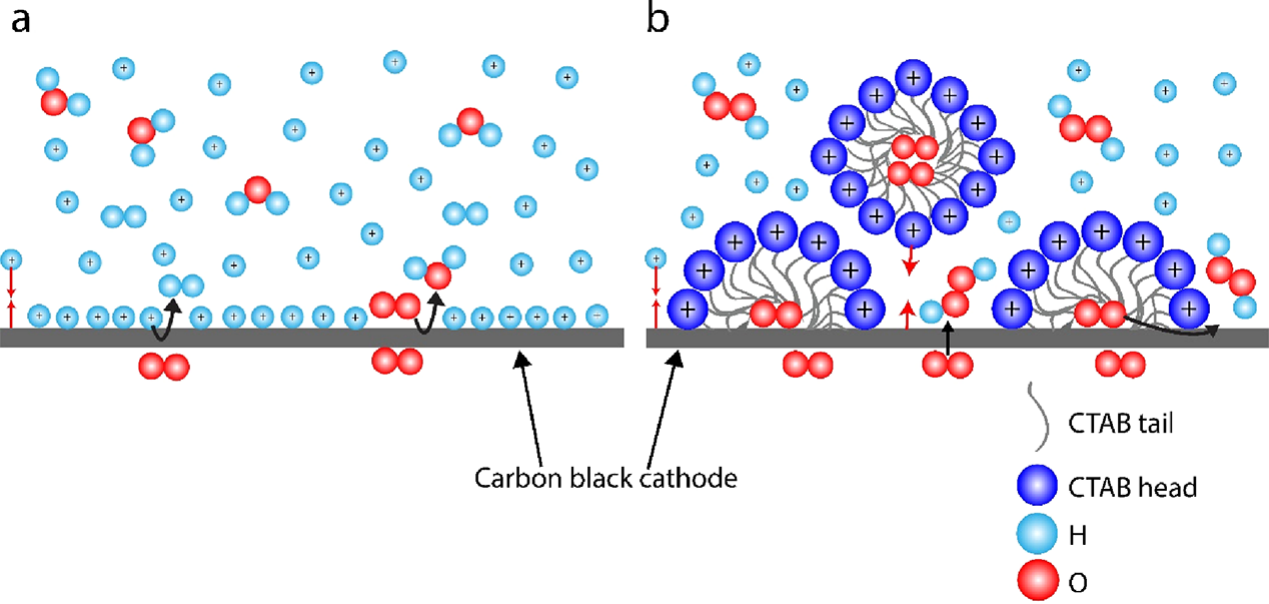
Schematic portraying the hypothetical EDL at the cathode interface (a) without CTAB and (b) with CTAB. H (cyan), CTAB tail (gray), CTAB head (blue), and O_2_ (red).

Inspired by biology, wherein micelles protect cells from the extracellular matrix, we propose utilizing amphiphilic surfactants to tune the hydrophobicity of the electrode–electrolyte interface and protect the electrode surface from the acidic bulk electrolyte.^21,22^ Micelles in solution may have either or both of two effects (Figure 1): increased O_2_ solubility and thus increased O_2_ transport due to micellization^23^ and a shielding effect that displaces protons from the EDL.^21,22,24^ To demonstrate this idea, we added cetyltrimethylammonium bromide (CTAB, Figure S1a), a commonly used surfactant, to a strong acidic electrolyte (pH ∼ 1) to evaluate its impacts on the ORR selectivity of the carbon black catalyst. Carbon black catalyst has been demonstrated to show excellent H_2_O_2_ selectivity in alkaline electrolyte but poor selectivity in acidic solutions. However, when CTAB was added to the acidic electrolyte, the carbon black catalyst showed excellent H_2_O_2_ selectivity (>90%) under significant ORR current (>200 mA cm^–2^). The enhancement effect from CTAB was also observed in the presence of other TAB surfactants, notably dodecyltrimethylammonium bromide (DTAB, Figure S1b) and hexyltrimethylammonium bromide (HTAB, Figure S1c). In situ SERS and in situ OM were applied to further investigate the surfactant effect. The SERS data reveals that CTAB interacts more strongly with the electrode at more negative potentials, which is reasonable given that TAB surfactants possess a positive charge. Additionally, in situ OM demonstrates that CTAB aggregates become immobilized as the reduction potential is increased. We attribute this effect to the adsorption of CTAB to the electrode surface. This study provides an interfacial engineering tool for enhancing H_2_O_2_ electrosynthesis as well as mechanistic insights into the underlying phenomena. The new strategy proposed and demonstrated in this paper can be further applied to develop other interfacial engineering techniques that can be used to scale up our electrolyzers for more practical applications.

## Results

### Electrochemical Reduction of O_2_ to H_2_O_2_ in an Acidic Environment Using a Flow Cell

**Figure 2:**
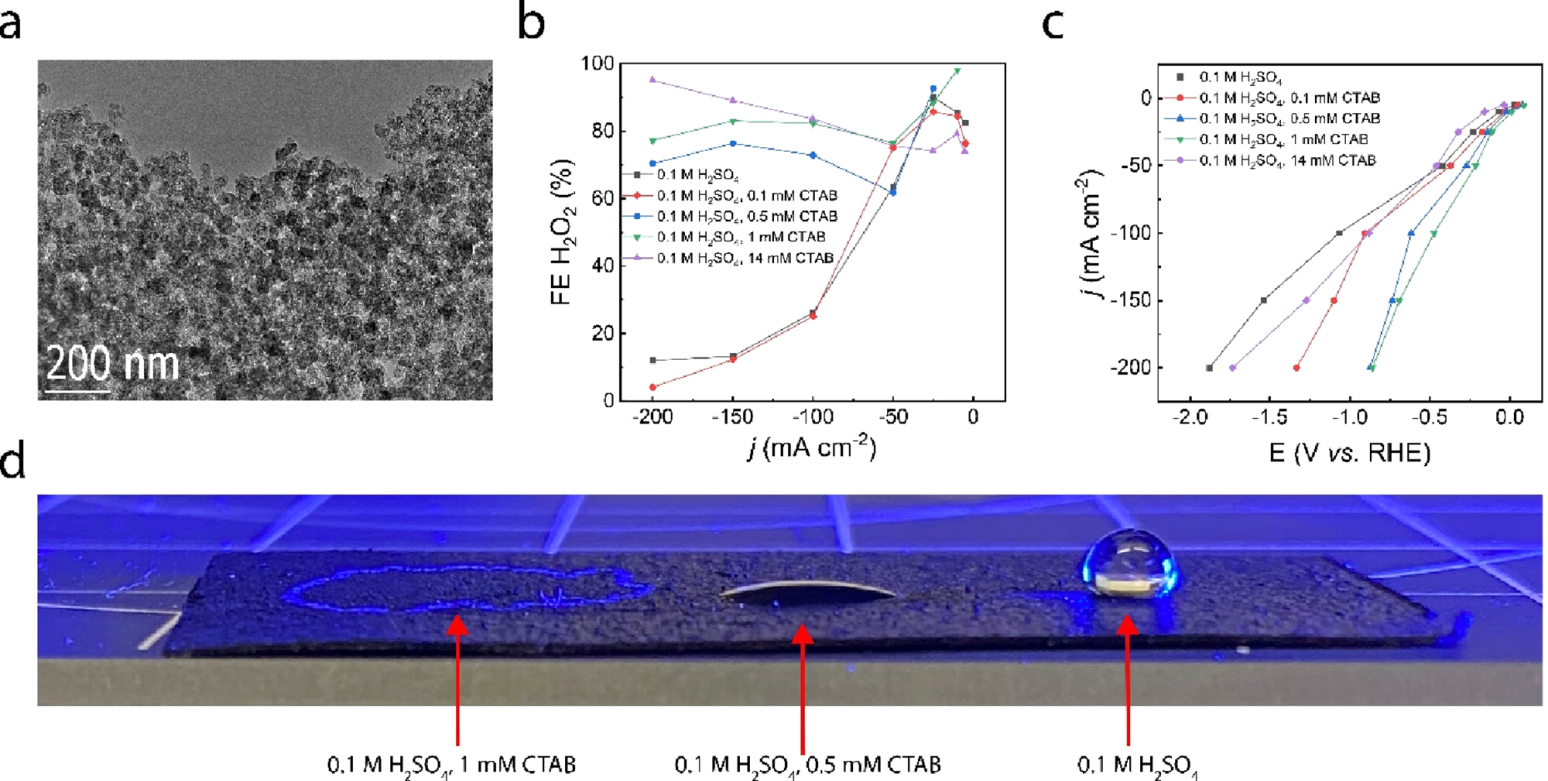
(a) TEM image of carbon black. Effect of CTAB concentration on (b) H_2_O_2_ selectivity and (c) electrocatalytic activity. (d) Contact angles of different electrolytes on the GDE.

In order to measure the effects of the surfactant systems on 2e^–^-ORR, we carried out electrochemical measurements in a standard three-electrode flow cell with a saturated calomel electrode (SCE) as the reference electrode. Carbon black (BP2000) was chosen as the ORR catalyst due to its low cost and high selectivity (Figure 2a), while commercial IrO_2_ was chosen as the anode catalyst. To maintain a low pH of ∼1, we used 0.1 M H_2_SO_4_ as the electrolyte. At low overpotentials, the selectivity of H_2_O_2_ on carbon black was above 80%, achieving a maximum selectivity of 90% H_2_O_2_ at a current density of 25 mA cm^–2^ and a potential of −0.23 V vs RHE. However, by 100 mA cm^–2^ and −1.07 V vs RHE, the selectivity had already dropped to 26% in pure sulfuric acid solution. The drop in the H_2_O_2_ FE with increasing overpotential is expected, as the cathode would accumulate more negative charges, therefore attracting more protons to the electrode–electrolyte interface. We then added varying concentrations of CTAB to the electrolyte, noting that the critical micelle concentration (CMC), the concentration of surfactant required for micellization, is ∼0.9 mmol L^–1^ (mM).^25,26^Figure 2b reveals that as the CTAB concentration increased so did the selectivity toward H_2_O_2_. Adding 0.5 mM CTAB to the electrolyte, for example, resulted in a selectivity of 76% FE H_2_O_2_ at a current density of 150 mA cm^–2^ and a potential of −0.74 V vs RHE, compared with 13% FE H_2_O_2_ without surfactant. With 1 mM CTAB added to the electrolyte, the FE further increased to 83% at 150 mA cm^–2^ under −0.69 V vs RHE. When 14 mM CTAB was added to the electrolyte, the cell delivered a peak FE of 95% H_2_O_2_ at a current density of 200 mA cm^–2^ and a potential of −1.73 V vs RHE. We posit that CTAB displaces protons near the electrode surface, precluding the further reduction of H_2_O_2_ and the HER. CTAB could be directed toward the electrode surface due to the Coulombic interaction resulting from its positive charge and the negative charge of the electrode under reduction conditions.^27^ Micellization of CTAB molecules could also help tune the hydrophobicity of the interface and favor H_2_O_2_ synthesis. To further confirm high H_2_O_2_ selectivities in the presence of CTAB, we measured the H_2_ selectivity at the cathode outlet using gas chromatography (GC). As observed in Figure S2, we detected no observable H_2_ peak, which would reflect a HER process occurring at the cathode. The only detectable peak was from unreacted O_2_. Additionally, we observed that the activity increased with increasing CTAB concentration (Figure 2c), although this relation is not true for the 14 mM CTAB sample, which may have been too concentrated for favorable performance. At high surfactant concentrations, the gas diffusion layer (GDL) is susceptible to flooding, which may irreversibly damage the electrode. The activity relation demonstrates that the ORR kinetics are more sluggish in the absence of surfactant.

The O_2_ transport depends on an aerophilic environment for fast and effective gas transport. In order to measure the hydrophobicity of the electrode–electrolyte interface, we calculated the contact angles of the electrolytes on the gas diffusion electrode (GDE) for varying concentrations of CTAB. As depicted in Figure 2d and Figure S3a and c, the contact angle of the pure acid electrolyte was 136°, compared with 68° in the presence of 0.5 mM CTAB and 0° in the presence of 1 mM CTAB. The 1 mM CTAB electrolyte clearly wets the GDE and can make the electrode more hydrophilic. At first glance, this result may appear to conflict with our electrochemical results, which reveal a high selectivity and activity of the 2-e^–^ ORR when CTAB is in solution. To compensate for the increased wetting of the GDE, we believe micellization could be a key factor for the increased O_2_ concentration at the electrode–electrolyte interface,^23^ in turn leading to higher activity and higher selectivity toward the H_2_O_2_ product. Thus, the increased hydrophilicity of the CTAB solution on the electrode surface does not have an adverse effect on the reaction, and the micelles are likely responsible for creating the aerophilic environment necessary for the ORR.

### In Situ Experiments to Understand Behavior at the Electrode–Electrolyte Interface

**Figure 3:**
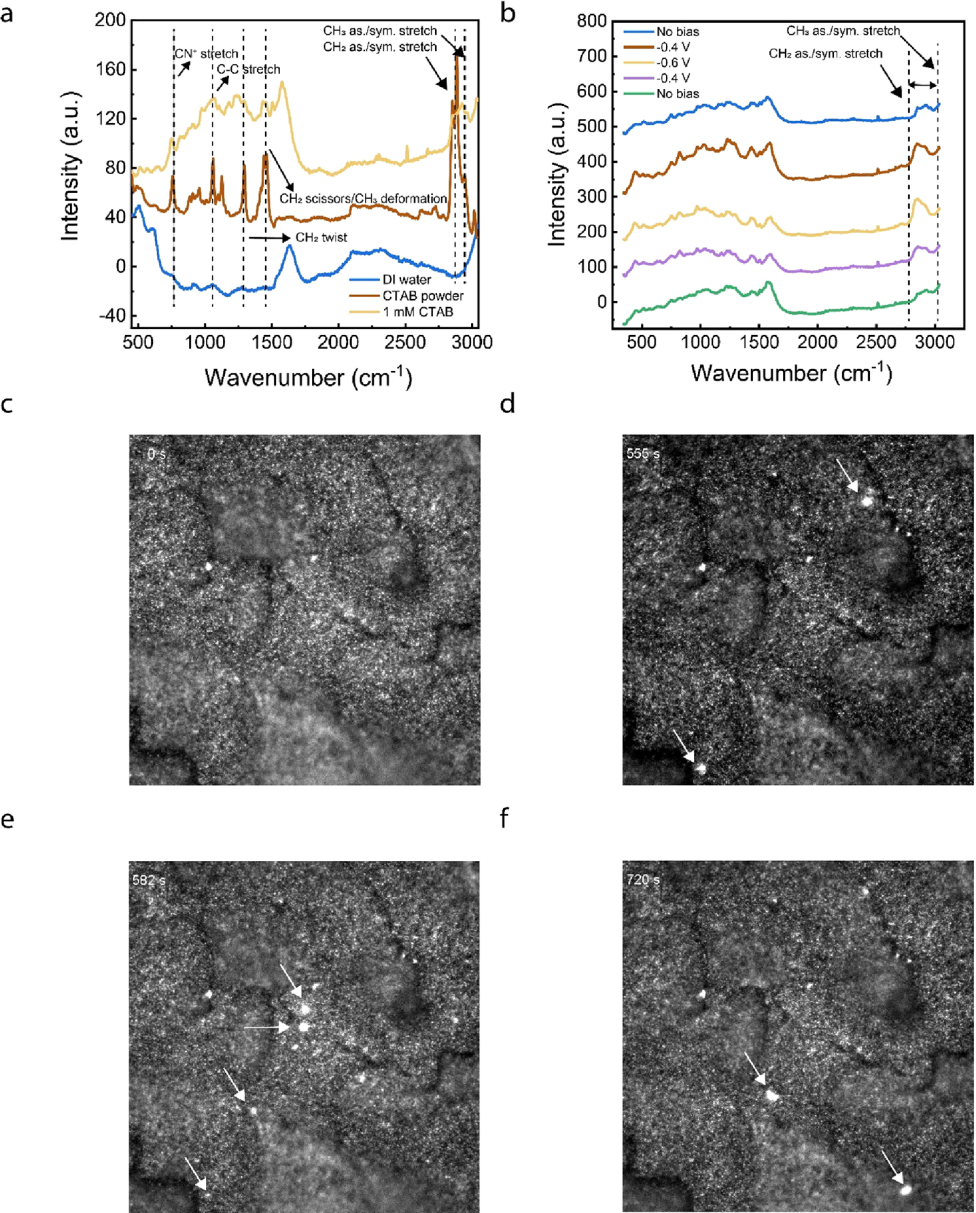
Raman spectroscopy data (a) of 0.1 M H_2_SO_4_ with 1 mM CTAB, CTAB powder, and DI water on Au foil with Au nanoparticle. Region 1 depicts CH_2_ scissoring, twisting, and wagging. Region 2 depicts CH_2_ stretching. (b) In situ SERS data at different potentials on Au foil with Au nanoparticles; (c–f) optical microscopy images of the CTAB micelle–electrode interactions at various timestamps during the activation process.

We performed in situ Raman measurements to understand the interactions between the CTAB molecules and the electrode surface. To enhance the Raman signals, we deposited Au nanoparticles (50 nm in diameter) onto a Au electrode (50 nm Au on a glass substrate). The yellow curve in Figure 3a shows the Raman response of 1 mM CTAB in 0.1 M H_2_SO_4_ in the absence of electrolysis. For comparison, the Raman signal in DI water was also plotted (blue curve in Figure 3a). Two broad Raman band regions have been observed. The first region, which is at low wavenumbers (between 700 and 1600 cm^–1^), includes CH_2_ scissoring and CH_2_ twisting/wagging modes.^28,29^ The second region between 2800 and 3000 cm^–1^ corresponds to the C–H stretching mode of CTAB.^28−32^ We also measured the Raman spectrum of CTAB powder (brown curve in Figure 3a), and the results reveal two broad Raman regions that are similar to those of the CTAB sample in solution (yellow curve in Figure 3a). This proves the observed Raman signal in the electrolyte is related to the CTAB molecules. Note that we see differences between the powder sample and the solution sample, which can be attributed to substrate effects and have been observed in other literature as well. In addition, the CTAB powder signals appear more intense compared with the respective signals in solution due to the higher concentration of the CTAB sample under the microscope. More importantly, the low frequency modes overlap with the strong substrate bands and are more difficult to resolve unambiguously.^28,29^ Therefore, we use the C–H stretching modes (between 2800 and 3000 cm^–1^) as the preferred region to detect and understand the CTAB–electrode interactions.

Figure 3b shows the Raman measurements conducted at different potentials in the 0.1 M H_2_SO_4_, 1 mM CTAB electrolyte. We started the experiment without any electric bias (blue curve in Figure 3b), and we clearly observed the C–H stretching mode of CTAB in the spectrum. This phenomenon indicates that the adsorption of CTAB onto the electrode surface can occur even in the absence of an electric potential. When we began to apply a negative potential (−0.4 V vs SCE, brown curve in Figure 3b), the intensity of the C–H stretching peak increased, indicating an enhanced adsorption of CTAB on the electrode surface. We believe this is due to the Coulombic attraction between the negatively charged electrode surface and the positively charged CTAB molecules. When the potential was further increased to −0.6 V vs SCE, the C–H stretching mode of CTAB increased again (yellow curve in Figure 3b). We then scanned the potential back to −0.4 V versus SCE (purple curve in Figure 3b), and the intensity of the peak decreased. Finally, the signal almost recovered to the original strength after the potential was removed (green curve in Figure 3b). This demonstration reveals that CTAB electrically adsorbs to the electrode surface under reduction potentials. While the adsorption strength is not too high to the point where CTAB irreversibly adheres, the CTAB adsorption does increase at larger overpotentials, providing insight into why the CTAB can help maintain a high H_2_O_2_ selectivity at large currents.

In a similar study, an optical microscope allowed us to visually observe the electrode–electrolyte interface directly on the carbon black electrode surface in real time. We used a transparent flow cell with 1 mM CTAB for visual purposes. Since the average size of a CTAB micelle is ∼5 nm in diameter,^33,34^ it is impossible to detect the individual CTAB micelle using an optical microscope. To view the CTAB micelle and substrate interactions, we increased the micelle concentration and introduced relatively large micelle aggregates that could be observed with an optical microscope. The carbon electrode and catalyst absorbed most of the light, and the reflected light intensity was minimized. The individual CTAB aggregates could serve as scattering centers and show up as bright spots in the images (Supplementary Videos S1–S8). We imaged the electrode surface during the entire 20 min of the activation process (Supplementary Video S1). At the beginning of the activation, the electrode surface appeared relatively clean, and we did not observe any CTAB aggregates (Figure 3c). Within 13 min of activation, the bright CTAB aggregates began to attach to the electrode surface (pointed by the white arrows in Figure 3d–f). After activation, we recorded the electrode surface at different electric potentials (Supplementary Videos S2–S8). At no bias, 0, and −0.5 V vs SCE, we witnessed the CTAB aggregates freely moving around the electrode surface and interacting with the electrode. At −1 and −2 V vs SCE, the CTAB aggregates moved toward the electrode and adhered to the electrode. At the same time, the CTAB aggregates were accumulated on the electrode surface. At −3 and −4 V vs SCE, there were almost no free CTAB aggregates in the imaging view, indicating the complete adhesion of CTAB to the electrode surface. The OM images provide strong and direct evidence to further support the idea that CTAB micelles adsorb onto the electrode surface, which would provide sufficient transport of the O_2_ for 2e^–^-ORR catalysis.

To ascertain how long the CTAB could remain on the electrode surface after electrochemical activation, we conducted a stability test, whereby, after activation at 150 mA cm^–2^ for 30 min, we switched the electrolyte from 1 mM CTAB in 0.1 M H_2_SO_4_ to the pure acid electrolyte (Figure S4) while continuing to monitor the H_2_O_2_ selectivity. The selectivity did not show an immediate drop to the performance level, which we observed in the pure acidic electrolyte test. In fact, the H_2_O_2_ FE remained above its value at 150 mA cm^–2^ in pure acid (13% FE H_2_O_2_) for over 2 h. With no continuous supply of surfactant solutions, we do not expect micellization to have had an impact on this lag in the drop in H_2_O_2_ selectivity. Instead, the effect can be attributed to CTAB molecules binding to the electrode surface, likely due to the negative charge of the cathode under electrolysis conditions.

### Double Layer Capacitance Measurements

For stronger evidence of the ability of CTAB to displace protons at the EDL, we analyzed the double layer capacitance (*C*_dl_) of the various electrolyte systems: pure H_2_SO_4_ and 0.5 and 1 mM CTAB in H_2_SO_4_. We calculated the *C*_dl_ from cyclic voltammetry curves (CVs) at varied scan rates (Figure S5a–d).^35^ We measured the *C*_dl_ in the range of 0.4–0.5 V vs RHE due to this being a non-Faradaic (negligible reaction) potential range. The results demonstrate that *C*_dl_ decreases with increasing CTAB concentration. We postulate that the CTAB decreases the charge accumulation at the EDL due to the long, hydrophobic chains of the molecule that hinder ion transport. The CTAB molecules thus have an ability to tune the hydrophobicity near the electrode surface and increase the local pH. This decrease in charge accumulation agrees with our claim that, despite the CTAB increasing the degree of wetting of the electrode, the interfacial concentration of protons is reduced. Moreover, the electrochemical active surface area (ECSA)-normalized activity of the catalyst operating under various CTAB concentrations improves with increasing CTAB concentration (Figure S6). This observation reveals that, even after accounting for the differences in ECSA, the intrinsic activity is further enhanced in the presence of CTAB.

### Extending the CTAB Effect to Other Surfactants

**Figure 4:**
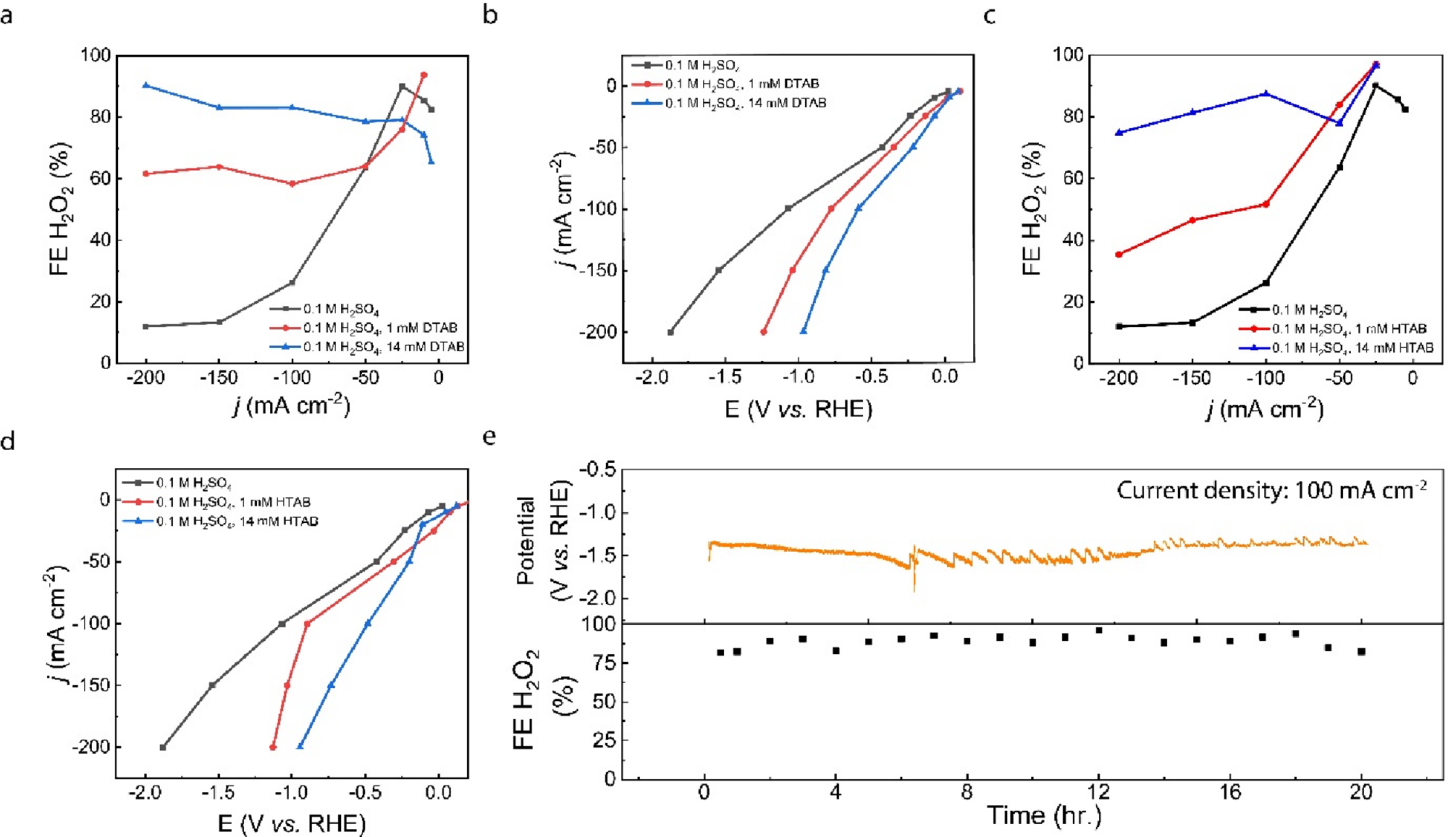
DTAB (a) H_2_O_2_ selectivity and (b) electrochemical activity. HTAB (c) H_2_O_2_ selectivity and (d) electrochemical activity. (e) Stability of 0.1 M H_2_SO_4_, 14 mm DTAB electrolyte in the flow cell. Current density was set to 100 mA cm^–2^.

To confirm whether the enhancement effect from CTAB could be extended to other TAB surfactants, we compared electrochemical results from HTAB (CMC ∼ 1 M)^25^ and DTAB (CMC ∼ 14 mM).^25,36^ Whereas CTAB has a maximum alkyl chain length of 16 carbons, HTAB and DTAB have maximum alkyl chain lengths of 6 and 12, respectively. An electrolyte consisting of 14 mM DTAB yielded an H_2_O_2_ FE of 90% at a current density of 200 mA cm^–2^ and a potential of −0.967 V vs RHE, nearly 1 V less energy input than the pure acid electrolyte at the same current density with a 7.5-fold increase in selectivity (Figure 4a,b). While HTAB requires 1 M surfactant to form micelles, we observed significant Br_2_ evolution while testing this highly concentrated electrolyte sample. The Br_2_ evolution reaction can occur from the crossover of Br^–^ to the anode, which very large concentrations of TAB surfactants can induce. These concentrations are not necessary, given that we can achieve >90% selectivity H_2_O_2_ with <0.02 M CTAB and DTAB. To illustrate this, we demonstrated that 14 mM HTAB could achieve 90% and 81% FE H_2_O_2_ at current densities of 100 and 150 mA cm^–2^, respectively (Figure 4c). The trend of increased selectivity and activity with increased surfactant concentration holds for HTAB and DTAB, as it does for CTAB (Figure 4a–d). Thus, the surfactant effect is universal to all TAB surfactants, and some enhancement can still be achieved without micelles present in solution.

We excluded the counteranion effect on the enhanced H_2_O_2_ activity and selectivity by changing the surfactant from CTAB to cetyltrimethylammonium chloride (CTAC). CTAC has the exact same molecular structure as CTAB, except with Cl^–^ as the counteranion instead of Br^–^. When CTAC was employed in our electrolyzer, a maximum H_2_O_2_ selectivity of 90% FE was achieved at 150 mA cm^–2^ and −0.75 V versus RHE (Figure S7). Finally, because industrial applications require long-term operation of electrochemical devices, we investigated the stability of the 14 mM DTAB electrolyte system. We chose this electrolyte sample due to its high H_2_O_2_ selectivity and high tolerance to GDE flooding. In the presence of 14 mM DTAB, the flow cell delivered >80% FE H_2_O_2_ for over 20 h (Figure 4e), revealing the high stability of the electrolyzer in the presence of surfactants.

## Conclusion

This work demonstrates an interfacial engineering approach for the electrochemical generation of H_2_O_2_ in a strongly acidic environment. An 8-fold increase in the H_2_O_2_ selectivity at 200 mA cm^–2^ resulted in 95% FE H_2_O_2_ in the presence of 14 mM CTAB when compared with the pure acid solution. Furthermore, the reaction kinetics were improved with the addition of surfactants to the electrolyte, and we demonstrated high stability of the system over 20 h. In situ Raman spectroscopy and OM showed that CTAB particles adsorb onto the electrode surface during electrolysis, rendering a more aerophilic environment for O_2_ transport, and in turn enhancing the H_2_O_2_ selectivity. *C*_dl_ measurements further confirmed the increased hydrophobicity and heightened pH with the addition of surfactants to the electrolyte, allowing for a selective 2e^–^-ORR process. While the results from this study are promising, more effort is required for this surfactant strategy to be implemented in the industrialization of acidic H_2_O_2_ electrosynthesis.

## Methods

### Materials

CTAB and Nafion perfluorinated resin solution (527084-25 mL) were purchased from Sigma-Aldrich. HTAB and DTAB were purchased from TCI. Cerium sulfate (Ce(SO_4_)_2_) was purchased from Alfa Aesar. The sulfuric acid and methanol were from Millipore Corporation. Isopropanol was purchased from VWR Chemicals. Carbon black (BP2000) was purchased from Cabot Corporation.

### Electrode Preparation

We prepared suspensions comprised of 40 mg of carbon black, 4 mL of 2-propanol, 1 mL of methanol, and 80 μL of Nafion binder. The suspension was sonicated in an ice bath for 30 min and then spray coated onto a 5 × 5 cm^2^ GDL (Sigracet 28 BC, Fuel Cell Store) using an Air Brush. To dry the catalyst, we put the electrode in a vacuum chamber for 12 h. For use as a cathode in our flow cell, we then cut the GDE into 0.5 × 2 cm^2^ pieces.

### Electrolyte Preparation

We prepared varying concentrations of electrolytes of CTAB, HTAB, and DTAB by dissolving calculated amounts of the surfactants into 0.1 M H_2_SO_4_ solutions. For preparation of the 0.1 M H_2_SO_4_ solution, we mixed 2.72 mL of concentrated H_2_SO_4_ with 497 mL of Millipore H_2_O (18.2 MΩ cm) for each 500 mL of electrolyte required.

### Nafion 117 Membrane Activation

Nafion 117 membrane (Fuel Cell Store) was activated prior to electrochemical testing for the best results. In order to remove organic impurities, we heated the membrane at 80 °C in 5 wt % H_2_O_2_ for 1 h. We placed the membrane into H_2_O again for 1 h at 80 °C to remove all residual H_2_O_2_. Next, we immersed the membrane in 1 M H_2_SO_4_ for 1 h at 80 °C to activate the membrane for H^+^ crossover. Lastly, we placed the membrane in 80 °C water for 1 h to fully clean it.

### Electrochemical Flow Cell Experiments

The flow cell utilized incorporated a saturated calomel electrode (SCE, CH Instruments) as the reference electrode. We used the following equation to convert experimental potentials versus SCE (*E*_SCE_) into RHE (*E*_RHE_): *E*_RHE_ = *E*_SCE_ + 0.244 + 0.0591 × pH. The cell consisted of an IrO_2_ anode (Fuel Cell Store) and a carbon black cathode, both 0.5 × 2 cm^2^, with Nafion PEM in between. The catalyst layers on both electrodes faced the middle electrolyte flow channels. We flowed 30 sccm O_2_ gas to the backside of the GDE via an Alicat Scientific mass flow controller (MFC). The catholyte and anolyte were both supplied to the cell via peristaltic pumps (Huiyu pump YZ15-13A), both operated at 2 rpm (∼1.8 mL min^–1^). We conducted all electrochemical experiments using a Biologic VMP3 workstation. We began the testing with resistance readings of the cell. This was conducted via a potentio electrochemical impedance spectroscopy (PEIS) measurement. The reported potentials in this study were all 80% compensated for the *iR* drop. After measuring the cell resistance, we carried out chronopotentiometry (CP) tests. We ramped the cell current up in 60 s steps: 1, 5, 10, 25, 50, and 100 mA to gradually ramp up the cell potential without damaging the electrode. After the energy reached 100 mA, we activated the catalyst for 20 min. Then, we returned the current to 5 mA, where we waited another 20 min before measuring the cell potential and collecting the H_2_O_2_ product for concentration measurement. This brief activation period ensured the maximum enhancement effect of the surfactant, as we noted small increases in selectivity during this time. The cathode outlet line was simultaneously rinsed well with DI water to remove highly concentrated H_2_O_2_. We measured selectivity and cell potential at the following currents: 5, 10, 25, 50, 100, 150, and 200 mA. All of the selectivity measurements reported are averages of two data points.

### Quantification of H_2_O_2_ Concentration

For measuring the H_2_O_2_ concentration in the catholyte outlet stream, we exploited the reaction of Ce^4+^ with H_2_O_2_.
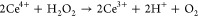
1Ce^4+^ possesses a yellow color, while Ce^3+^ is colorless. We measured the Ce^4+^ concentration in a solution after reaction with the H_2_O_2_ product through use of a UV–vis spectrophotometer (UV-2600, Shimadzu).^14,15,37^ Ce^4+^ has an absorption peak at ∼317–319 nm. In order to react Ce^4+^ with H_2_O_2_, we combined 50 μL of H_2_O_2_-containing solution with 4 mL of 0.5 mM Ce(SO_4_)_2_. To maintain a stable Ce(SO_4_)_2_ solution, we dissolved Ce(SO_4_)_2_ in 0.5 M H_2_SO_4_. When H_2_O_2_ is dropped into 0.5 mM Ce(SO_4_)_2_, the solution loses some of its yellow color, making UV–vis detection an easy measure of Ce^4+^ concentration. We determined the Ce^4+^ concentration from a calibration curve based on 0.1, 0.2, 0.3, 0.4, and 0.5 mM Ce(SO_4_)_2_ solutions in 0.5 M H_2_SO_4_ (Figure S8). Then, we utilized eq 2 to determine the H_2_O_2_ concentration, based on the reaction in eq 1,
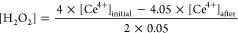
2where all concentrations in brackets are in mmol L^–1^. FE was then determined from eq 3
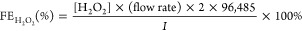
3where *I* is the current in mA, the flow rate is in mL s^–1^, 96,485 is the Faraday constant (C mol^–1^), and the H_2_O_2_ concentration is in mol L^–1^. This method was used for measuring the H_2_O_2_ concentration only for currents up to 150 mA, as we used the faster KMnO_4_ titration method for currents above 150 mA. We explain why Ce^4+^ titration was required below.

The KMnO_4_ titration is derived from the following chemical reaction:
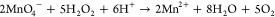
4

We employed a standard KMnO_4_ solution (0.1 N, Sigma-Aldrich), and 1 M H_2_SO_4_ was the H^+^ source. The concentration of H_2_O_2_ in the product stream was determined based on the volumes of H_2_O_2_-containing solution and KMnO_4_ at the titration end point, where the solution color turned from purple to clear. Equation 3 was then used for the selectivity calculation. While this titration method is faster and easier than the previous method, we found that at low currents, where low concentrations of H_2_O_2_ were generated, the surfactant in the electrolyte sample influenced the titration end point. The Ce^4+^ titration process, however, was not affected by the presence of a surfactant in solution.

### Quantification of H_2_ Gas

We measured the H_2_ concentration using GC (Shimadzu GC-2014 GC).

### Double Layer Capacitance Measurements

We conducted cyclic voltammetry (CV) experiments for measurement of *C*_dl_ of the electrode under various conditions. The bounds of the CV were 0.4 and 0.5 vs RHE, with a start and end point of 0.45 V. For each surfactant condition tested, we carried out 5 cycles of CVs. The following scan rates were used: 2, 4, 6, 8, and 10 mV s^–1^.

### In Situ Surface Enhanced Raman Spectroscopy and Optical Microscopy

For in situ SERS measurements, we dropped 50 nm-diameter Au nanoparticles on a Au foil electrode, which was then placed on a glass substrate so the laser could strike the sample. We dropped a 1 mM CTAB solution on top. The counter electrode was a Ag wire, and the reference electrode was the SCE. All electrodes were dipped into the electrolyte. The schematic of the home-built Raman microscopy system is shown in Figure S9. The home-built confocal Raman microscopy module was coupled with an inverted optical microscope (Olympus IX83). A diode laser beam at 642 nm (Vortran Stradus) was used to focus on the electrode surface and to excite the Raman scattering signal. The 20× water immersion objective (working distance: 2 mm, N.A. 1.0, Thorlabs) was applied to collect the Raman scattering signal and bright-field images. After separation with a spectrometer (iHR550, Horiba), the Raman signals were recorded by a charge-coupled device (CCD) for further analysis. We observed the Raman spectra from 500 to 3000 cm^–1^.

For the OM videos, we constructed a transparent 3-electrode cell, again with Ag wire as the counter electrode and SCE as the reference electrode. The working electrode was the same as that in the electrochemical experiments with the flow cell, a GDE with carbon black coated on it. The cell contained one compartment, but we incorporated a divider to ensure no liquid electrolyte would contact the upward-facing backside of the GDE. The dry backside of the GDE ensured O_2_ could penetrate the GDL. We transported O_2_ to the backside of the GDE via a gas line, and all excess O_2_ was free to disperse via an outlet gas line. A white light source (Thorlab) was used to illuminate the sample, and a scientific CMOS camera (Hamamatsu, C11440-42U30) was applied to record bright-field images and videos, with a spatial resolution of ∼300 nm.

## Supporting Information Available

Molecular structures, contact angles, UV–vis calibration curve, in situ microscopy schematic, and additional data (PDF)
Supplementary Video S1 - Electrode–electrolyte interface during the 20 min activation period (AVI)
Supplementary Video S2 - Electrode–electrolyte interface after the 20 min activation process (AVI)
Supplementary Video S3 - Electrode–electrolyte interface under 0 V vs SCE (AVI)
Supplementary Video S4 - Electrode–electrolyte interface under −0.5 V vs SCE (AVI)
Supplementary Video S5 - Electrode–electrolyte interface under −1 V vs SCE (AVI)
Supplementary Video S6 - Electrode–electrolyte interface under −2 V vs SCE (AVI)
Supplementary Video S7 - Electrode–electrolyte interface under −3 V vs SCE (AVI)
Supplementary Video S8 - Electrode–electrolyte interface under −4 V vs SCE (AVI)

## Supplementary Material

pc3c00096_si_001.pdf

pc3c00096_si_002.avi

pc3c00096_si_003.avi

pc3c00096_si_004.avi

pc3c00096_si_005.avi

pc3c00096_si_006.avi

pc3c00096_si_007.avi

pc3c00096_si_008.avi

pc3c00096_si_009.avi
