# Supplementary material for: Hydrogen Peroxide Electrosynthesis in a Strong Acidic Environment Using Cationic Surfactants
Source: Precis Chem. 2024 Feb 3;2(4):129–37. doi: 10.1021/prechem.3c00096 (PMC11504663; doi:10.1021/prechem.3c00096)
Supplement: Supplementary file 1 — pc3c00096_si_001.pdf [file pc3c00096_si_001.pdf]

Supporting Information

Hydrogen peroxide electrosynthesis in a strong acidic environment using cationic surfactants

Zachary Adler[1], Xiao Zhang[1], Guangxia Feng[2], Yaping Shi[2], Peng Zhu[1], Yang Xia[1], Xiaonan Shan[2*], and Haotian Wang[1,3,4*]

Rice University, Department of Chemical and Biomolecular Engineering, Houston, TX 77005, USA

University of Houston, Department of Electrical and Computer Engineering, Houston, TX 77004, USA

Rice University, Department of Chemistry, Houston, TX 77005, USA

Rice University, Department of Materials Science and Nanoengineering, Houston, TX 77005, USA

*Corresponding authors:

Haotian Wang htwang@rice.edu (H.W.)

Xiaonan Shan xshan@central.uh.edu (X.S.)

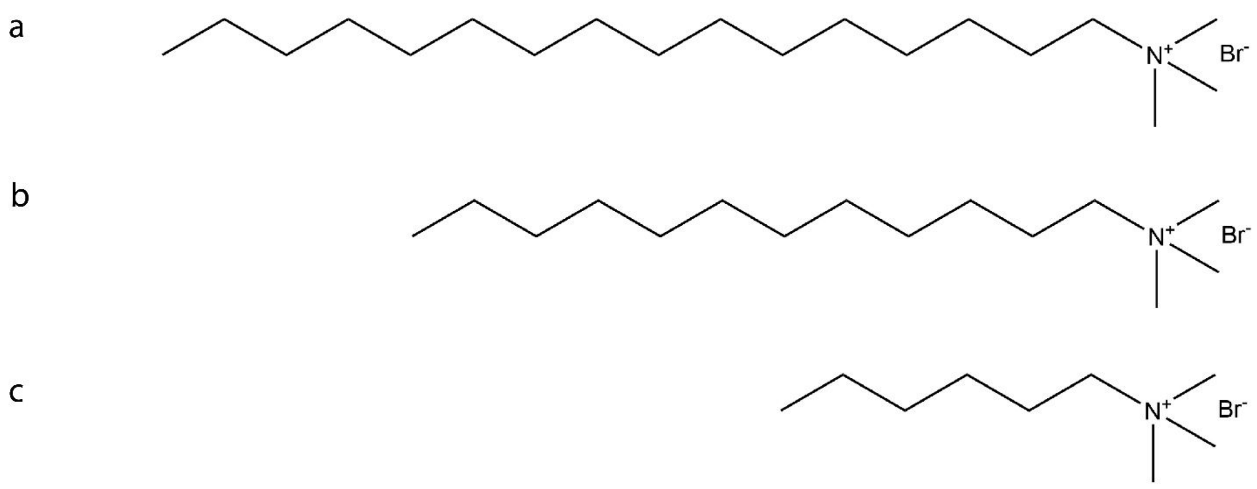

Figure S1. Molecular structures of a.) CTAB, b.) DTAB, and c.) HTAB.

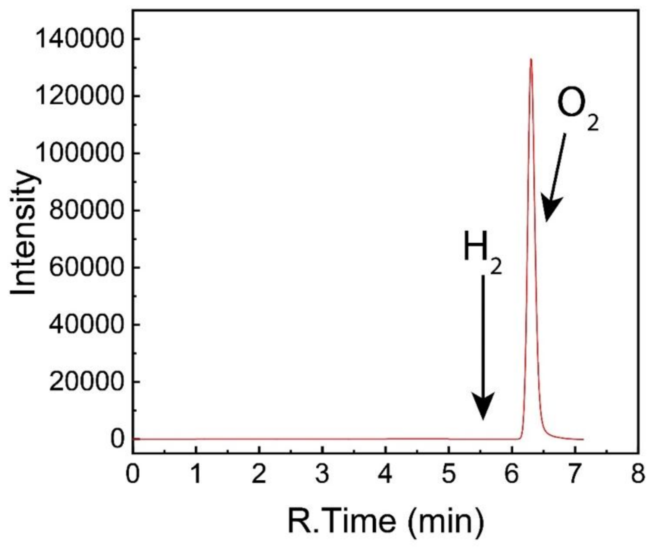

Figure S2. GC spectrum of the 1 mM CTAB sample at 200 mA cm^{-2}.

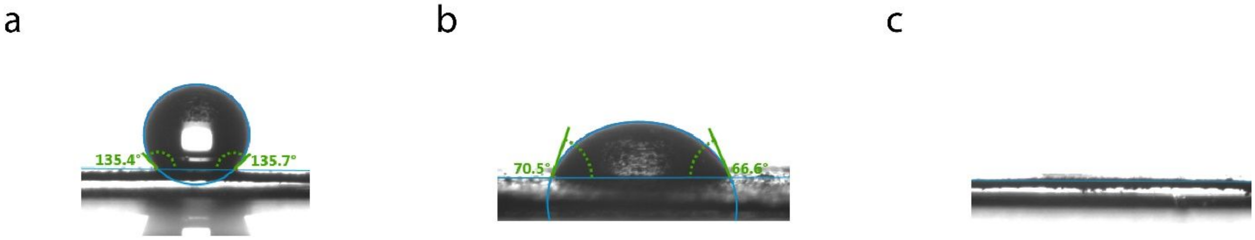

Figure S3. Contact angles of a.) 0.1 M H_2SO_4, b.) 0.5 mM CTAB in 0.1 M H_2SO_4, and c.) 1 mM CTAB in 0.1 M H_2SO_4 on the carbon black GDE.

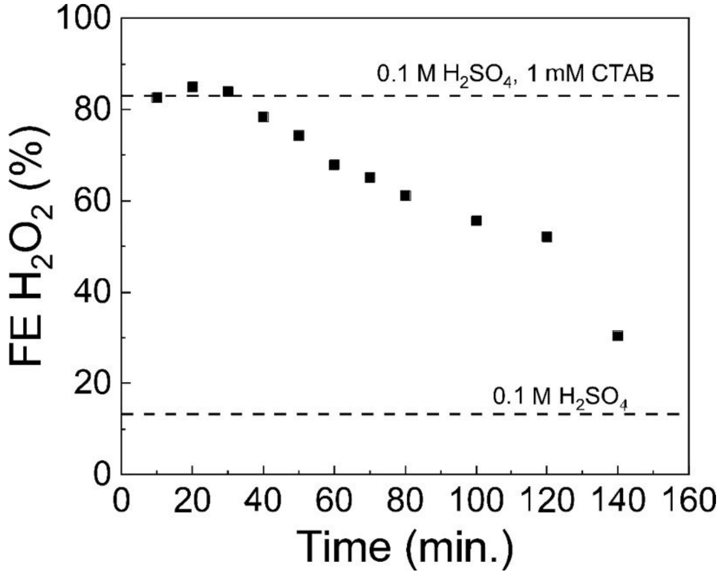

Figure S4. Stability of pure acid electrolyte at 150 mA cm^{-2}. The cell was previously activated at 150 mA cm^{-2} in 0.1 M H_2SO_4, 1 mM CTAB for 30 min.

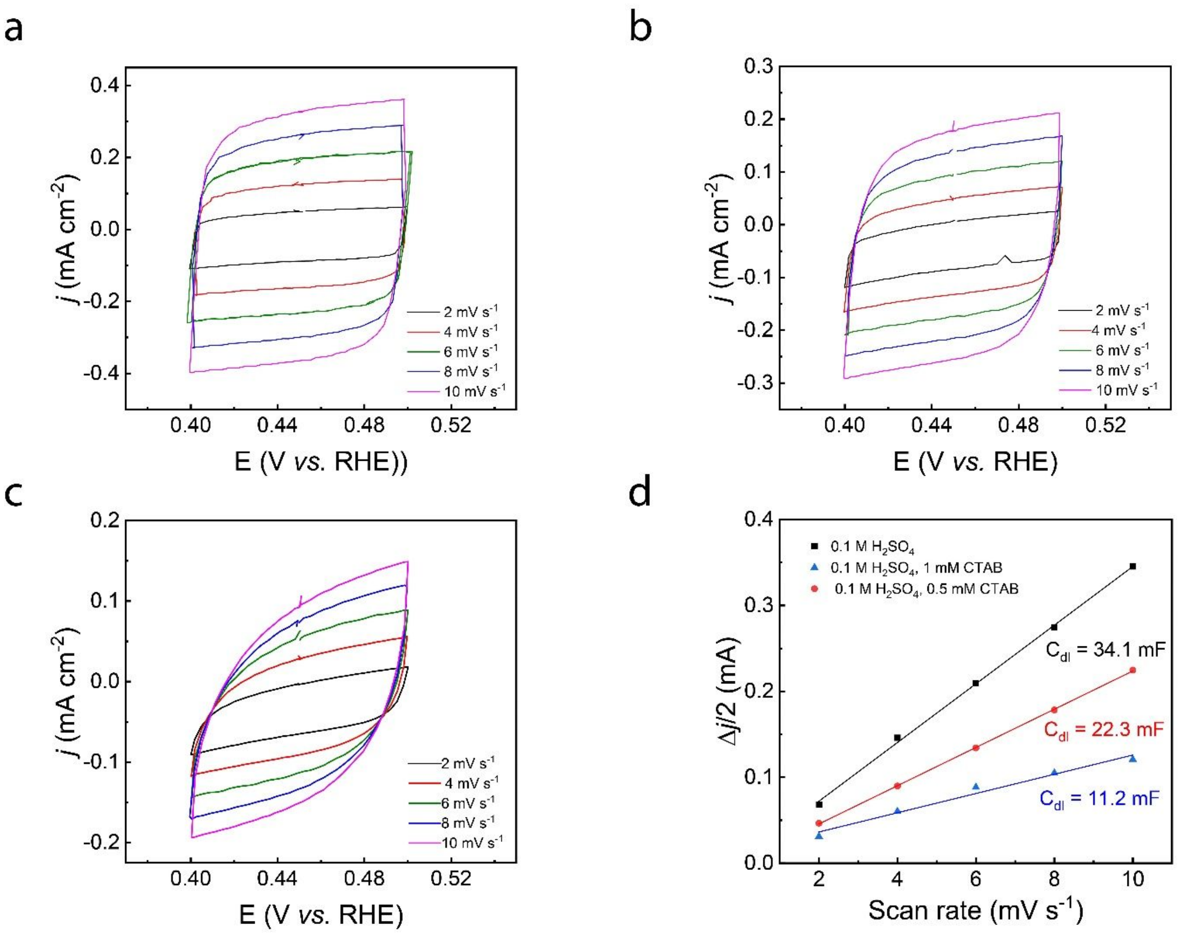

Figure S5. C_{dl} measurements. a-c.) Cyclic voltammetry curves of 0 mM CTAB, 0.5 mM CTAB, and 1 mM CTAB, respectively. d.) We plotted the electrochemical scan rate against the differences of the halves of the positive and negative current densities after 5 cycles of CV at 0.45 V vs RHE. The C_{dl} was derived from the slope of the resulting line.

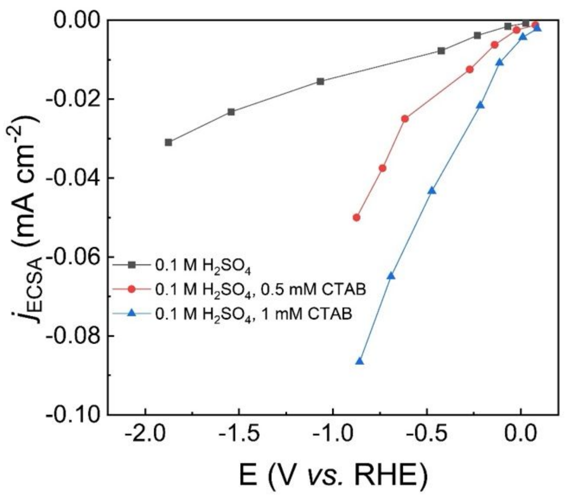

Figure S6. Electrochemical surface area (ECSA)-normalized current density versus potential.

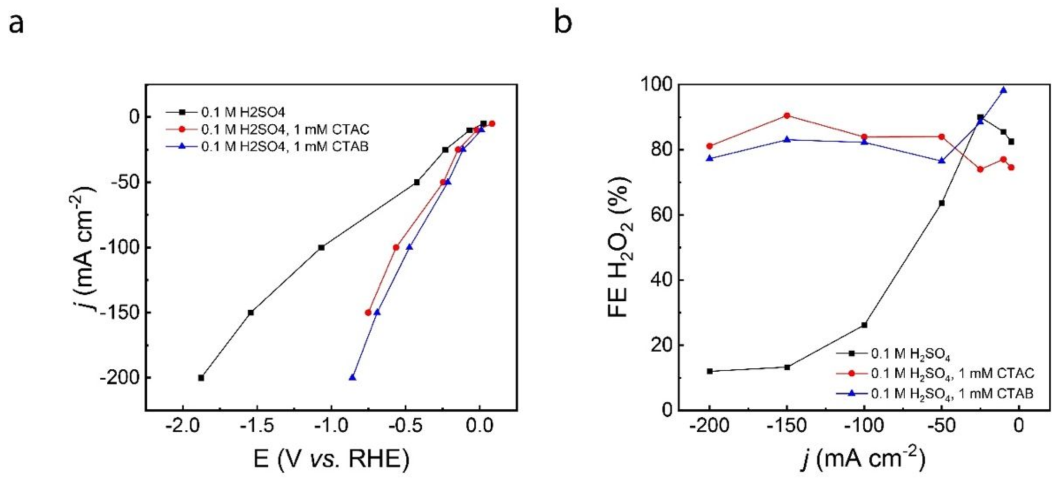

Figure S7. Comparison of CTAB and CTAC. a.) Electrochemical activity and b.) H_2O_2 selectivity.

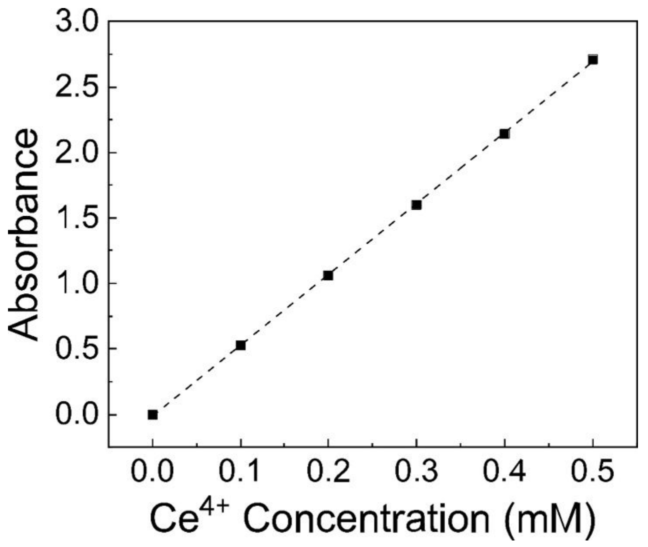

Figure S8. UV-vis. calibration curve used for measurement of Ce^{4+} concentration.

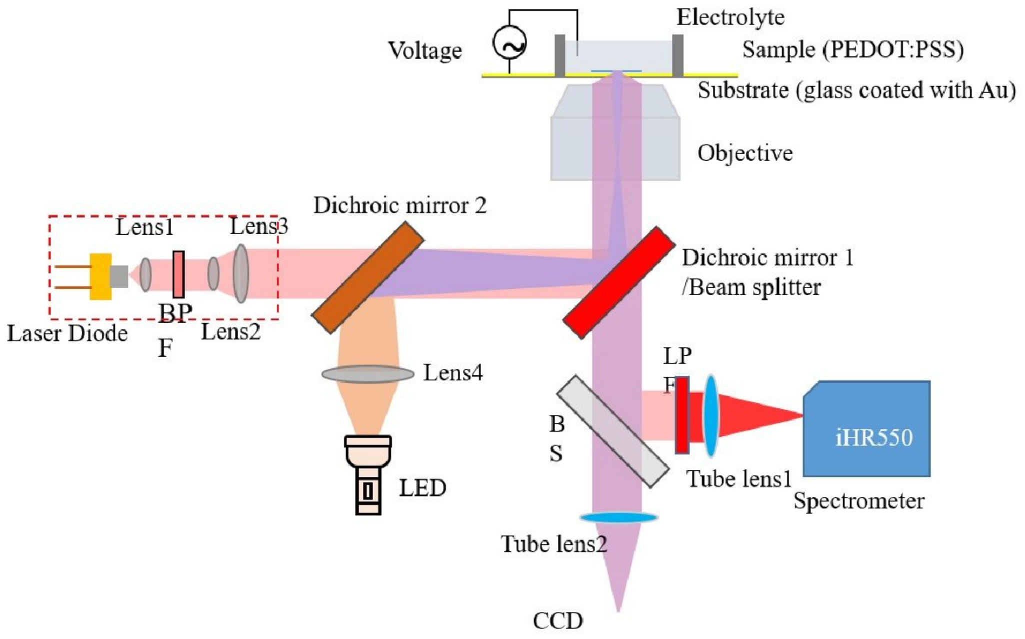

Figure S9. Schematic of the in-situ Raman spectroscope and optical microscope.
